# The Impact of Pressure Training on the Performance of Semi-Professional Female Rugby League Players

**DOI:** 10.3390/bs14090856

**Published:** 2024-09-23

**Authors:** Michele Lastella, Sharni Upton, Dean J. Miller

**Affiliations:** 1 Appleton Institute, Central Queensland University, Adelaide, SA 5034, Australia; d.j.miller@cqu.edu.au; 2 School of Health, Medical and Applied Sciences, Central Queensland University, Rockhampton, QLD 4700, Australia; sharni.upton@cqumail.com

**Keywords:** stress, rugby, team sport, performance, passing

## Abstract

Background: The aim of this study was to examine the effect of pressure training on the performance of semi-professional female rugby league athletes. Methods: Using a within-subjects design, 16 female athletes (19.9 ± 3.4 years) performed a passing accuracy task under three conditions; (1) a control condition; (2) a physiological fatigue condition; and (3) a threat of consequence condition. Passing performance, perceived pressure, rate of perceived exertion (RPE), and self-confidence were assessed. Results: A significant main effect of conditions was found for rate of perceived exertion (*p* < 0.001), self-confidence (*p* < 0.028), and perceived pressure (*p* = 0.011). There was no main effect of condition on passing performance. Post hoc comparisons revealed that RPE was significantly higher in the physiological fatigue condition when compared to the control (*p* = 0.009) and threat of consequence conditions (*p* < 0.001). Perceived pressure was significantly higher in the threat of consequence condition compared to the control condition (*p* = 0.037). Conclusions: The main findings of this study are that (1) passing performance was not impacted by pressure training conditions, and (2) threats of consequences are an effective manipulation to generate pressure in female semi-professional rugby league players. These results offer nuanced insights into the impact of pressure generation in training environments for female semi-professional rugby league athletes.

## 1. Introduction

As part of the training process, coaches and practitioners frequently try to replicate the demands of competition by implementing pressure during training. Pressure training has been conceptualised as an individual process or part of a multimodal intervention delivered with additional performance-related training [1]. Researchers have applied various theoretical frameworks to pressure training, including transactional stress and coping, theory of challenge and threat states, and attentional control theories [2]. Pressure training resembles traditional cognitive behavioural interventions such as systematic desensitisation and stress inoculation training [1,2,3]. 

Pressure training has gained popularity among coaches for familiarising athletes with various forms of pressure [4,5]. While pressure training may take many forms, for example, pressure inurement training [6], anxiety training [7], acclimatisation training [8], or stress exposure [9], it ultimately seeks to enhance performance by practicing skills under simulated pressure [1]. Despite the evidence that pressure training may improve performance across various sports [4,10,11,12,13], there are limited data examining the application of pressure in training environments [2]. 

In the context of generating pressure and fatigue within training environment, research has consistently found that consequences or fear of consequences are effective [11,12]. Stoker et al. [12] observed that consequences, such as peer evaluation and forfeits, induced pressure among elite netballers, while demands alone did not seem to generate the same level of pressure. Furthermore, Lyons et al. [14] found athletes with higher skill levels exhibited superior performance under conditions of fatigue compared to novice or less skilled athletes.

At present, there is a need for research on the pressure training processes, relevance, and functionality in different task and competition environments [3]. Like most areas of sport science, data related to pressure training has primarily focused on male athletes compared to female athletes [13], making the application of specific pressure manipulations across different sports and populations challenging [5]. This is critical as several physiological differences related to hormones and muscle strength may affect how pressure training translates into performance gains and injury risk [15,16]. Taking into consideration how previous data has demonstrated that pressure training interventions improve performance outcomes across various sports [4,10,12,13], it is important to address the limited knowledge base pertaining to pressure training in female athletes. Therefore, the aim of this study was to (1) examine the extent that physiological fatigue and threat of consequence can produce pressure in semi-professional female rugby league athletes, and (2) examine the effects of these conditions on skill execution and athletes’ confidence. 

## 2. Materials and Methods

### 2.1. Study Design

A repeated measures design was utilised to investigate the effect of three conditions (control, physiological fatigue, and threat of consequence) on three performance variables (skill execution, perceived pressure, and self-confidence). In all conditions, participants completed a passing accuracy test and provided ratings of perceived pressure and self-confidence. In the control condition, participants completed the task without any manipulation or intervention. In the physiological fatigue condition, participants completed the task after performing a three-minute repeated physical conditioning task. In the threat of consequence condition, participants were told that they would be required to do an additional physical conditioning task if they failed to improve their passing accuracy scores, compared to the control and physiological fatigue conditions. 

To maintain the integrity of the independent variables, counterbalancing was not employed, as the perception of the threat of consequence condition may be influenced by the physiological fatigue condition. Therefore, the order of the conditions (control, physiological fatigue, threat of consequence) was designed to progressively increase the task difficulty for each condition, to mitigate potential practice effects [17]. This study was conducted in accordance with the Declaration of Helsinki and approved by the Central Queensland University Human Research Ethics Committee (Ethics number: 2023-029) for studies involving humans.

### 2.2. Participants

Sixteen female semi-professional rugby league players (mean age ± standard deviation; 19.9 ± 3.4 years) volunteered to participate in the present study (Table 1). Participants had playing experience at a semi-professional level (mean playing experience; 5.3 ± 2.9 years). Among player roles, there were 8 outside backs (N = 8), 6 forwards categorised as middle forwards (N = 3) and edge forwards (N = 3), while there was one hooker (N = 1) and half, respectively (N = 1). The majority of participants (94.8%) were of right-hand dominant passing preference. 

### 2.3. Procedures

Data were collected over three separate nights at the team’s indoor training facility. Participants were given verbal instruction of the requirements of the study and reminded of their right to withdraw at any time. Participants provided written informed consent and completed a general health questionnaire to acquire demographic information. 

On each experimental night, participants completed their usual team warm-up, stretching, dynamic movements, and passing. Participants then presented individually and were then given instructions on how to complete the passing accuracy test. The passing accuracy test involved ten stationary rugby league passes towards a circular target from two set distances (four and seven metres). At each distance, participants performed five passes from both the left and right passing directions. Immediately following the completion of the 10 passes at each distance, participants reported their perceived pressure, confidence, and rate of perceived exertion experience via a numerical scale. For the control and threat of consequence conditions, the passing accuracy test commenced one-minute after the completion of instructions. In the physiological fatigue condition, participants commenced the passing accuracy test one-minute after completion of the maximal aerobic speed (MAS) conditioning drill. Data from the passing accuracy tests were collected on separate nights for each condition, but in a fixed order (i.e., control, physiological fatigue, and threat of consequence).

To control for additional pressure variables, observation was limited to the student researcher, strength and conditioning coach, and one coaching staff member. To ensure that no additional physiological demand was created, participants were instructed to not collect the balls after passing. Each participant was requested to not discuss their experiences with other participants until the completion of the study. This process was repeated across each condition. Data collection was performed over a 3-week period, with 7 days between each condition.

## 3. Materials

### 3.1. Maximum Aerobic Speed 

The maximum aerobic speed (MAS) running task involved alternating between running and resting periods every 15 s, aiming to sustain a high intensity sprint during the running segments [18]. The specific distances for each participant were determined individually based on their established training protocols, as advised by the club’s strength and conditioning coach.

### 3.2. Passing Accuracy Test 

**Figure 1:**
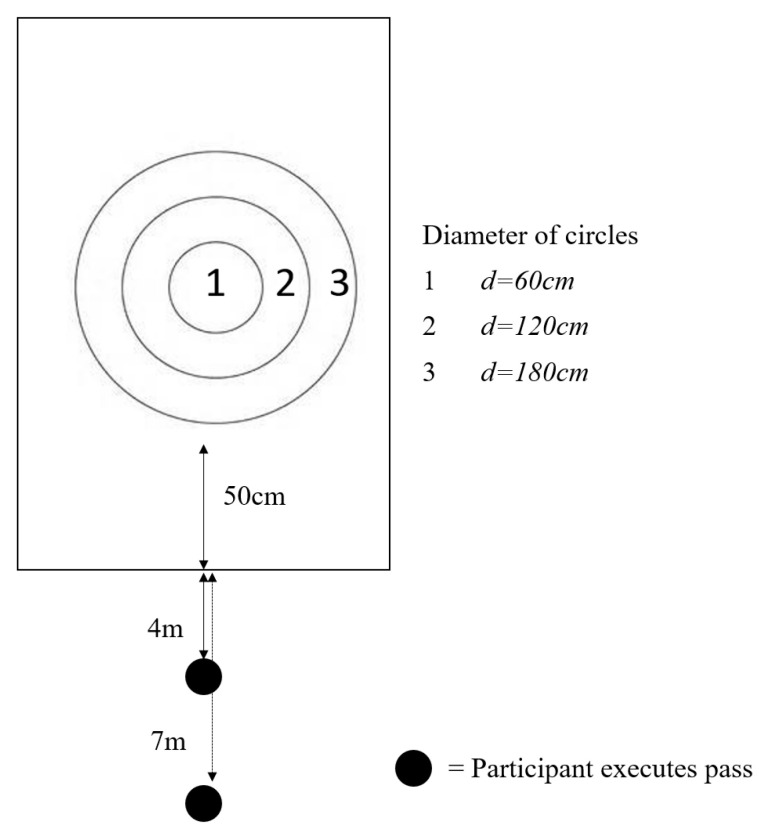
Visual representation of the passing accuracy test described in Hendricks et al. [19].

Passing performance was measured with a passing accuracy test that was assessed at two set distances (four metres and seven metres) across each condition (Figure 1) [19]. The passing accuracy test has previously been validated by Pienaar et al. [20]. Participants were required to pass a standard size 5 rugby league ball from a stationary position at a fixed target from two distances (four metres and seven metres). Participants were provided with five attempts at passing from both left and right side across both distances. Higher scores reflected higher pass accuracy, with points allocated depending on where the pass landed in relation to the circles (innermost circle = 3 points, middle circle = 2 points, outmost circle = 1 point, pass fails to land in a circle = 0 points; Figure 1). Total passing accuracy scores were calculated by combining points from the left and right passing sides, from both distances in each condition (maximum score of 30).

### 3.3. Self-Reported Confidence and Perceived Pressure Experience

To determine perceived pressure, participants were asked to report pressure as a numerical value ranging from one to one hundred (i.e., “please rate the pressure you experienced during this task on a scale of 0–100, with “0 = no pressure and 100 = maximum pressure”). Participants reported perceived pressure after completing the passing test at the 4 m distance and 7 m distance. Participants average perceived pressure scores were collected for each condition. 

To determine confidence in skill execution, participants were asked to report confidence as a numerical value ranging from one to one hundred (i.e., “please rate the level of confidence you experienced during this task on a scale of 0–100”, with “0 = no confidence and 100 = maximum confidence”). Participants responded after completing the passing test at the four metres distance and seven metres distance. 

### 3.4. Rating of Perceived Exertion

To determine the rating of perceived exertion (RPE), participants subjectively rated their level of exertion while performing the passing accuracy test at each distance using the rating of perceived exertion Scale [21]. Participants reported their rates of exertion as a numerical value ranging from six “no exertion at all” to twenty perceiving a “maximal exertion of effort”. 

### 3.5. Data Analyses

Data were analysed using IBM SPSS Statistics (v24.0; IBM Corp., Armonk, NY, USA) with data presented as mean ± SD. Statistical significance was determined using an α level of 0.05 and effect sizes were calculated using partial eta squared (η^2^) and interpreted as small (0.01), medium (0.06), and large (0.14). The distribution and sphericity of data were assessed via the Shapiro–Wilk test and Mauchly’s test of sphericity. Separate repeated measures ANOVAs were completed for the dependent variables (RPE, perceived pressure, self-confidence, and performance), with conditions (control, physiological fatigue, threat of consequences) as the within-subjects factor. The a priori power analysis (G*Power 3, Heinrich Heine University Dusseldorf, Dusseldorf, Germany) determined that 16 participants would be needed to reach significance with a power of 0.961 at an alpha level of 0.05. Where necessary, post hoc analyses were conducted with Bonferroni adjustments.

## 4. Results

### 4.1. Rated Perceived Exertion 

The physiological fatigue condition produced the highest average RPE score, followed by the control condition, and the threat condition produced the lowest average RPE score (Table 2). There was a significant main effect of condition on RPE, such that the physiological fatigue condition resulted in significantly higher RPE when compared to the control condition (*p* = 0.009) and threat of consequence condition, respectively (*p* = 0.001). 

### 4.2. Passing Performance 

There was no main effect of condition on passing performance. The control condition produced the highest passing accuracy scores, followed by threat of consequence and physiological fatigue (Table 2). However, there was no main effect of condition on passing performance.

### 4.3. Reported Pressure 

Participants felt the least confident during the threat of consequence condition, followed by the physiological fatigue condition, and then the control condition (Table 2). There was a main effect of condition on perceived pressure, such that the threat of consequence condition produced significantly higher reported pressure when compared to the control (Table 2; *p* = 0.037). *p* = 0.011, η^2^ = 0.261.

### 4.4. Reported Confidence 

A one-way repeated measures ANOVA was conducted to determine whether there was a statistically significant difference in participants self-reported confidence across pressure conditions. The pressure condition produced a significant main effect on participants’ confidence during the passing accuracy test, F(2,30) = 4.020, *p* = 0.028, η^2^ = 0.211. Reported confidence was lowest in the threat of consequence condition compared to the physiological fatigue and control condition (Table 2). Post hoc analysis with a Bonferroni adjustment revealed no statistically significant differences in confidence between any of the three conditions (*p* > 0.05).

## 5. Discussion

This aim of this study was to examine how physiological fatigue and the threat of consequences create pressure, and how this pressure affects skill execution and confidence in semi-professional female rugby league athletes. The main findings were that (1) the threat of a consequence effectively generates pressure during skill execution tasks, and (2) the threat of a consequence and physiological fatigue did not impact reported confidence or passing accuracy.

### 5.1. Perceived Pressure

The findings of the present study indicated that there was a considerable increase in participants’ reported pressure in the threat of consequence condition compared with the physiological fatigue and control condition. The higher amount of perceived pressure in the threat of consequence condition finding was consistent with the previous research demonstrating the pivotal role consequences have in eliciting pressure [10,12,22]. For example, Stoker et al. [10] observed that perceptions of pressure, anxiety, and heart rate were higher among elite netballers when judgement, forfeit, and reward consequences were introduced to skill tasks. 

In the present study, the effective implementation of a threat of consequence as a pressure manipulation took the form of a negative physical consequence, with participants believing they were required to complete a maximal aerobic capacity (MAS) conditioning exercise if their passing accuracy did not improve. The fixed order of conditions ensured that each participant completed the MAS conditioning exercise in the second condition, preceding the threat of consequence condition. This sequence heightened participants’ motivation to perform well to avoid the consequence. The principles in this design supported the recommendations of Low et al. [5] that the threat of consequence should be timed to maximise the relevance of the consequence. 

The present findings support the notion that demand stressors in isolation are insufficient for generating pressure, as highlighted in previous research [12,22]. The results strengthened the argument that the creation of pressure must be distinguished from the inherent difficulty of a task, as the latter does not necessarily enhance the incentive to perform well [5]. Interestingly, previous data proposed that the introduction of additional demands may lead to less perceived pressure compared to situations where athletes were expected to perform well without added demands [15]. This may explain the lack of differences observed in the physiological fatigue condition yielding similar mean passing accuracy scores to the threat of consequence condition but failing to produce a significant increase in perceived pressure [23,24].

### 5.2. Passing Performance 

Ratings of perceived exertion were significantly higher during the physiological fatigue condition compared to the control and threat of consequence conditions. Findings revealed that participants’ passing accuracy results were not impacted by the physiological fatigue condition such that no differences were observed in passing scores between conditions. The finding that performance outcomes (i.e., passing accuracy) were not affected under perceived pressure is indicative of the complexity of simulating competition game situations [25]. It is plausible that performance outcomes under pressure may be mitigated by pressure familiarisation (i.e., when individuals have previously been exposed to similar pressure situations). 

Investigations into the efficacy of consequence and demand stressors have produced favourable effects on performance among athletes [7,26]. For example, Bell et al. [26] implemented a negative consequence model (e.g., cleaning the dressing rooms) during a leadership program to enhance coping skills among elite cricketers. Performance outcomes, including mental toughness, were positively impacted under pressure situations [26]. In contrast, Stoker et al. [12] found that passing accuracy in elite netballers remained unaffected by consequences such as judgement, forfeit and rewards. Instead, performance was reduced when exposed to task and environmental demands, including time constraints, visual occlusion, and noise distractions. Furthermore, Ötting et al. [27] examined elite dart players over a year-long period and found that pressure (characterised as game-winning opportunities) did not impact performance. Yet, Stoker et al. [23] found that demand stressors and judgement significantly decreased shooting performance in elite disability shooters. The inconsistent findings across studies may be attributed to differences in expertise and experience of athlete samples [22]. For example, the elite netballers in Stoker et al. [12] were accustomed to pressure training and had international experience, whilst Ötting et al. [27] used professional dart players with high levels of skill. While the youth cricketers utilised by Bell et al. [26] were familiarised with coping strategies.

In the context of the present study, it is possible that previous exposure to competition pressure allowed them to maintain performance under simulated pressure. The conditions selected in this study replicated what could be considered the most prevalent form of pressure in a rugby league match. Physiological fatigue, in which players must perform under physical fatigue, is a type of performer stressor that all players experience regardless of their playing position [23,24]. Furthermore, players must continuously perform under the threat of consequences. For example, if a player fails to execute a skill such as a poorly executed pass, it may result in a favourable outcome for the opposition. Consequently, it is feasible that each participant has previously encountered physiological fatigue and the threat of consequence in competitive scenarios. However, a noteworthy challenge in making direct comparisons is that a significant portion of pressure application research focuses on judgement and forfeit consequences, as well as task and environmental demand stressors.

### 5.3. Reported Confidence

There was a significant main effect of condition on reported confidence showing a difference in participants’ self-confidence scores. However, post hoc comparison did not reveal any statistically significant differences across the conditions. Observations of mean scores indicated that like the generation of pressure, threat of consequence produced the lowest confidence ratings, followed by physiological fatigue. The findings in the current study align with those of Stoker et al. [12,22], as both studies revealed a main effect for self-confidence intensity. However, like the present findings, post hoc comparisons in previous studies failed to observe any differences. Notably, the mean confidence scores observed in the current study conflicted with a previous study by Stoker et al. [12,22] such that mean confidence and performance scores were lower in conditions where demand stressors were increased. This was in contrast with the present study which demonstrated that participants’ mean confidence scores were lowest under the threat of consequence condition, corresponding with the highest reported pressure levels. 

It is worth noting the variation in confidence measurement methods. Stoker and colleagues [12,22] employed the well-established and validated shortened immediate anxiety measurement scale (IMAS), which is known for assessing state cognitive anxiety, somatic anxiety, and self-confidence [28]. In contrast, the present study used a numerical self-report scale to assess confidence. It is plausible that this difference in the measurement approach may account for disparities in the reported confidence scores across conditions between these studies.

### 5.4. Limitations

This study was conducted on a small number of semi-professional female rugby players (N = 16). The findings may not be generalised to other codes of football or professional rugby players as the demands or skill levels may differ. While the selection of a closed skill task like the passing accuracy test offers advantages in terms of replicability, it is important to acknowledge it assesses a discrete closed skill, characterised by relatively few movements. Thus, it may not replicate the complexity of passing in a rugby league game. In a competition situation, passing involves multidirectional interactions, including coordination with teammates and responses to defenders’ actions as cues of when to pass [29].

## 6. Conclusions

In short, the findings of the present study provide insights into the pressure training framework, particularly regarding the significance of forfeit consequences in generating pressure among female semi-professional female rugby league athletes. This contributes existing knowledge regarding pressure generation, thus offering coaches a range of effective consequence stressors for use in training. The study highlights the impact of pressure conditions, such as the threat of consequences and physiological fatigue, on participants’ self-rated confidence. However, it remains unclear how physiological fatigue and the threat of consequences distinctly influence athlete confidence. While this study did not find support for demand stressors in shaping performance, it does suggest the potential for demand stressors to complement consequence stressors effectively. It is essential to note that these findings may be limited to the specific participant sample, necessitating further research on the distinct roles of consequence and demand stressors in pressure generation and their effects on performance and self-confidence. In this context, the present study contributes to a better understanding of the systematic creation of pressure for effective pressure training.

## Figures and Tables

**Table 1:** Participant demographics.

Participant Demographics		Mean	SD
Age (Years)		19.9	3.4
Playing Experience (years)		5.3	2.9
		N	%
Playing Position			
	Outside Back	8	50.0
	Middle Forward	3	18.8
	Edge Forward	3	18.8
	Half	1	6.3
	Hooker	1	6.3
Experience playing other sports			
	Yes	13	81.3
	No	3	18.8
Other sports played			
	Touch Football	7	43.8
	Netball	3	18.8
	Basketball	1	6.3
	Oztag	1	6.3
Dominant Passing hand			
	Right	15	93.8
	Left	1	6.3

**Table 2:** Statistical results of female semi-professional rugby league athletes completing a PAT across pressure conditions.

	Condition			Statistical Outcomes		
	Control	Physiological Fatigue	Threat of Consequence			
	M ± SD	M ± SD	M ± SD	*F*(*df*)	*p*-Value	η^2^
RPE	11.50 ± 3.29	14.91 ± 2.85	10.66 ± 2.72	18.505(1.3, 19.6)	<0.001	0.552
Passing Performance	45.50 ± 6.69	43.62 ± 7.55	43.69 ± 6.82	1.675(2, 30)	0.204	0.100
Reported Confidence	53.25 ± 23.75	47.12 ± 20.41	39.69 ± 20.00	4.020(2, 30)	0.028	0.211
Reported Pressure	43.12 ± 21.88	53.22 ± 21.28	59.44 ± 18.43	5.305(2, 30)	0.011	0.261

## Data Availability

The data generated and/or analysed in the present study are available from the corresponding author on reasonable request.
